# Predictive significance of lymphocyte level and neutrophil‐to‐lymphocyte ratio values during radiotherapy in cervical cancer treatment

**DOI:** 10.1002/cam4.6221

**Published:** 2023-06-16

**Authors:** Mengli Zhao, Zhongrong Gao, Xiaowei Gu, Xiaojing Yang, Shanshan Wang, Jie Fu

**Affiliations:** ^1^ Department of Radiation Oncology Shanghai Sixth People's Hospital Affiliated to Shanghai Jiao Tong University School of Medicine Shanghai China; ^2^ Department of Radiation Oncology Jiangyin Hospital Affiliated to Nantong University Jiangyin China

**Keywords:** cervical cancer, lymphocyte, neutrophil‐to‐lymphocyte ratio, radiotherapy

## Abstract

**Objective:**

The objective of this research was to analyze the prognostic significance of the minimum count of lymphocytes (LY) and the corresponding ratio of neutrophil‐to‐lymphocyte (NLR) in cervical cancer (CC) patients receiving radiotherapy.

**Methods:**

We retrospectively collected data from 202 CC patients who received concurrent chemoradiotherapy or radiotherapy alone at our hospital. Statistical methods including the Kaplan–Meier method, log‐rank test and the Cox proportional hazards model were included to examine survival differences and identify independent factors that may affect overall survival (OS) and progression‐free survival (PFS).

**Results:**

The research enrolled a total of 202 patients. Patients with higher LY levels and lower NLR values during radiotherapy had significantly better survival prognosis than those with lower LY levels and higher NLR values. Multivariate COX regression analysis revealed that FIGO stage I, pathological types of SqCC, absence of lymph node metastasis, concurrent chemoradiotherapy, higher LY levels during radiotherapy, and lower NLR values before radiotherapy were independently associated with poorer PFS. Similarly, FIGO stage I, absence of lymph node metastasis and lower NLR values during and before radiotherapy were independently linked with poorer OS.

**Conclusion:**

Minimum LY value and its corresponding NLR during radiotherapy serve as prognostic factors for CC.

## 1 INTRODUCTION

Cervical cancer (CC) remains the fourth most prevalent type of malignancy among women, despite a significant reduction in incidence and mortality rates due to the widespread use of vaccines and advances in treatment techniques. According to data, around 600,000 new cases were reported in 2020 globally, with approximately 340,000 recorded deaths.^1^ Common management strategies for CC include surgery, radiation, and chemotherapy. Surgical excision is recommended for early‐stage tumors, whereas radical chemoradiotherapy is recommended for locally advanced patients. Radiotherapy is frequently used as a postoperative treatment to lower the risk of recurrence and metastasis.^2^, ^3^, ^4^ Clinical stage, histologic grade, lymphovascular space invasion (LVSI), lymph node metastasis, and extent of tumor infiltration are now considered the primary prognostic factors for the progression of CC.^5^ In addition to imaging and invasive tests, identifying predictive biomarkers can provide guidance for risk stratification and subsequent treatment.

Numerous researches have demonstrated the pivotal role of the tumor microenvironment in the occurrence, progression, metastasis, and prognosis of the cancer, and that inflammation is a crucial component of this environment.^6^, ^7^ Cancer patients with inflammation have been shown to respond less favorably to treatment and have poorer survival outcomes than those without inflammation.^7^, ^8^ Thus, inflammation‐related indicators have become a popular topic in cancer research, and several potential inflammatory prognostic indicators have been identified, including the platelet‐to‐lymphocyte ratio (PLR), neutrophil‐to‐lymphocyte ratio (NLR), lymphocyte‐monocyte ratio (LMR), and C‐reactive protein (CRP). It has been reported that NLR and blood indicators based on other inflammatory factors are effective predictors of prognosis in various solid tumors,^9^, ^10^ including endometrial cancer,^11^ gastric cancer,^12^ ovarian cancer,^13^ and CC.^14^, ^15^ However, recent research has primarily focused on the hematologic markers related to the inflammation on the prognosis of CC before treatment, with few scholars concentrating on the prognostic impact of inflammatory factors during treatment. Therefore, it is uncertain how inflammatory variables would influence the prognosis of CC following treatment.

In this study, representative hematological results during treatment were selected as research objects to generate more valuable research results. Because radiation induces lymphocytopenia, the lowest value of lymphocytes (LY) during radiation therapy may better reflect the immune status of the body during radiation therapy. Hence, we conducted this retrospective investigation to examine the predictive value of the minimum LY value and corresponding NLR value during radiotherapy on the prognosis of patients with CC. Meanwhile, LY and corresponding NLR values in hematological reports within 1 week before radiotherapy were collected and compared with those during radiotherapy.

## 2 MATERIALS AND METHODS

### 2.1 Patient selection

We reviewed clinical data on patients with primary cervical cancer in Stages I through IV of FIGO 2009, who underwent radiation therapy or concurrent chemoradiotherapy at our facility between June 2008 and September 2018. All patients underwent surgical treatment prior to radiotherapy, mainly consisting of radical hysterectomy and pelvic lymph node dissection. Radiotherapy was performed within 3 months after surgery. Patients who did not undergo surgery before radiotherapy or received other additional treatments alongside surgery, those with immune dysfunction, hematologic disease, severe hepatic or renal insufficiency, lack of pathological diagnosis, incomplete radiotherapy plan, incomplete blood parameter results, combined with second tumor, or who were lost follow‐up were excluded. In total, 202 patients with Stage I–II were enrolled, and clinical data were collected, including age, FIGO stage, pathological type, and lymph node status. Lymphocyte and neutrophil counts were collected during the 1 week prior to radiotherapy, as well as lymphocyte minimums and corresponding neutrophil values during the entire radiotherapy period, and the ratio of NLR was calculated.

### 2.2 Treatment

All enrolled patients underwent external pelvic irradiation by intensity modulated radiation therapy (IMRT). The average external radiation dose was 50.4 Gy, administered in proportions of 1.8–2.0 Gy. Weekly cisplatin (40 mg/m^2^) was the primary treatment regimen for patients who received synchronous chemotherapy, with three to five cycles depending on the treatment response. Some patients received brachytherapy and adjuvant chemotherapy after external irradiation.

### 2.3 Evaluation and follow‐up

Complete blood counts were measured weekly in all patients during radiotherapy. Follow‐up was carried out approximately every 3 months for the initial 2 years following the end of therapy, every 6 months for the following three to 5 years, and annually thereafter. In addition to being reviewed in our hospital, some patients were reviewed in their local hospitals for various reasons. Follow‐up methods included outpatient, inpatient, and telephone appointments. If imaging studies suggested recurrence or metastasis of the tumor, a biopsy of the affected area was conducted to verify the diagnosis. The most recent follow‐up date was in December 2022. The time from initial treatment to the last follow‐up, or the occurrence of relapse or death was defined as progression‐free survival (PFS). Meanwhile, the time from initial treatment to the last follow‐up or death was defined as overall survival (OS).

### 2.4 Statistical analysis

IBM SPSS software (version 26.0) was utilized in this research for the analysis of statistical. The predictive value of LY and NLR for prognosis was analyzed by ROC curve analysis, and the optimum cutoff value and corresponding specificity and sensitivity were obtained by using the maximum Youden index (sensitivity + specificity‐1) as the boundary. After employing the Kaplan–Meier to compute the survival disparity among groups, the log‐rank test was utilized to identify any significant variations between the groups. We carried out single‐variable and multiple‐variable analyses using COX proportional hazard models and examined 95% confidence intervals (CI) and hazard ratios (HR).

## 3 RESULTS

We reviewed the medical records and laboratory reports of 202 patients who met our inclusion standard and received radiation therapy at our facility. The patients' features are presented in Table 1, the median age of patients was 50 years (range, 24–77 years) at the time of treatment. Of all patients, 63% had Stage I and 37% had Stage II, with Stage IB1 and IIA being the most common, accounting for 42% and 30% of the total, respectively. Squamous cell carcinoma (SqCC) was the most prevalent histology type (88%), with 20 adenocarcinomas, 4 adeno‐squamous cell carcinomas, and 1 clear cell carcinoma. All patients accepted IMRT, with a median dose of external radiation of 50.4 Gy (range, 45–66.4 Gy) delivered in 1.8–2.0 Gy per proportion. In some patients, the local radiation dose was increased in the operative area, vaginal stump, or lymph node drainage area. Of the 117 patients (58%) who received concurrent chemoradiotherapy, 107 (53%) received concurrent cisplatin chemotherapy weekly for three to five cycles, while other concurrent chemotherapy regimens included docetaxel/platinum (eight patients), paclitaxel/platinum (two patients), and the remaining patients received radiotherapy alone. Some patients also received paclitaxel or docetaxel along with platinum‐assisted chemotherapy and brachytherapy.

**TABLE 1:** Synopsis of patient characteristics.

Variable	*N* = 202
Age at diagnosis, median (range)	50 (24,77)
Stage, *n* (%)	
Stage I	
IA	2 (1)
IB	
IB1	85 (42)
IB2	36 (18)
IB3	4 (2)
Stage II	
IIA	60 (30)
IIB	15 (7)
Pathology, *n* (%)	
SqCC	177 (88)
Other^a^	25 (12)
Lymph node, *n* (%)	
N (−)	150 (74)
N (+)	52 (26)
Treatment, *n* (%)	
CCRT	
Cisplatin alone	107 (53)
Other^b^	10 (5)
EBRT	85 (42%)
EBRT dose, median (range)	50.4 (45,66.4)

a Adenocarcinoma (20 patients), adenosquamous (4 patients), clear CC (1 patient).

b Docetaxel/platinum (8 patients), Paclitaxel/platinum (2 patients).

Abbreviations: CCRT, concurrent chemoradiotherapy; EBRT, external body irradiation.

For all follow‐up period time, the median time was 71 months. By the end of follow‐up, 33 patients had been passed away, and the median OS duration was 78 months (with a range of 10–175 months). The OS rates for 3‐ and 5‐year periods, as well as PFS, were 93%, 89%, 85%, and 74%, correspondingly. We analyzed the value of stage, histology, lymph nodes, treatment strategy, LY, and NLR for clinical outcomes in patients with CC. Patients with FIGO II and lymph node metastases had poorer PFS and OS, while patients with tissue squamous cell carcinoma and concurrent chemoradiotherapy had better PFS. Histological type of squamous cell carcinoma and concurrent chemoradiotherapy showed beneficial effects on PFS but had no significant effect on OS. We also measured the median LY and NLR values of patients before and during radiotherapy. The median LY and NLR values before radiotherapy were 1.5 × 109/L and 2.24, respectively, while during radiotherapy, the values were 0.4 × 10^9^/L and 5.2, respectively. The lowest lymphocyte count during radiotherapy ranged from 0.1 × 10^9^/L to 1.5 × 10^9^/L, with the majority of patients (83%) experiencing it during the third to fifth week of radiotherapy.

### 3.1 ROC curves of LY and NLR before and during radiotherapy for OS and cutoff values

**FIGURE 1:**
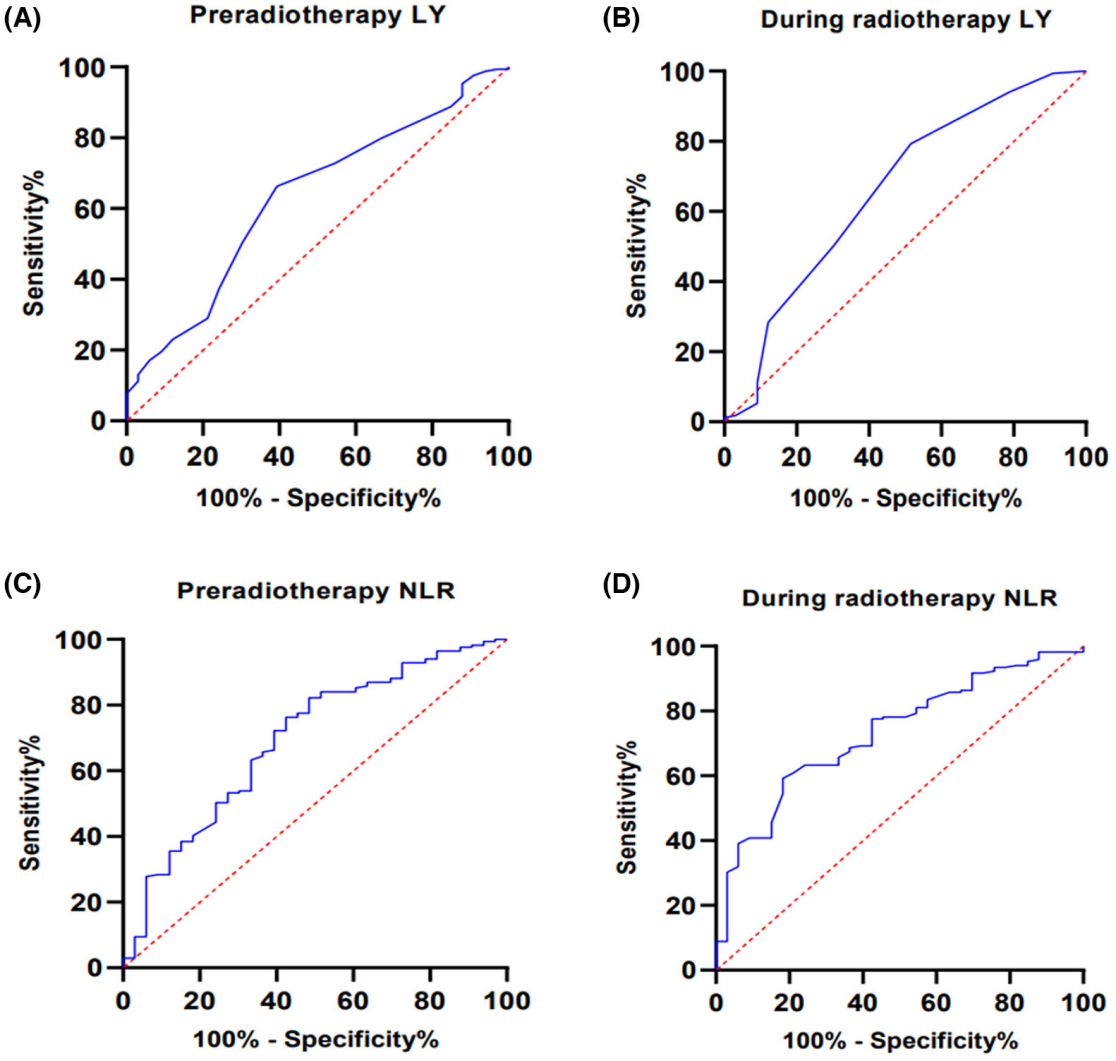
Shows the ROC curve analysis of LY and NLR values for overall survival in CC patients. (A) The ROC curve analysis of LY prediction before and during radiotherapy. (B) The ROC curve analysis of NLR prediction before and during radiotherapy.

Figure 1A,B presents the ROC curves utilized to detect the predictive significance of pre‐radiotherapy and during radiotherapy LY values for OS. The AUCs of pre radiotherapy and during radiotherapy LY were 0.630 (95% CI 0.528–0.733) and 0.662 (95% CI 0.552–0.772) (*p* < 0.05), respectively. The cutoff values were 1.45 × 10^9^/L for pre radiotherapy LY (sensitivity, 66.3%; specificity, 60.6%), and 0.35 × 10^9^/L for during radiotherapy (sensitivity, 79.3%; specificity, 48.5%).

Figure 1C,D shows the ROC curves utilized to detect the predictive significance of before radiotherapy and during radiotherapy NLR values for OS. The AUCs of pre radiotherapy and during radiotherapy NLR were 0.694 (95% CI 0.593–0.795) and 0.732 (95% CI 0.646–0.819) (*p* < 0.01), respectively. The cutoff values were 3.029 for pre radiotherapy NLR (sensitivity, 60.6%; specificity, 72.2%) and 5.071 for during radiotherapy NLR (sensitivity, 81.8%; specificity, 59.2%). These results indicate that LY and NLR values are significant indicators in forecasting the prognosis of CC.

### 3.2 Analysis of relationship between LY, NLR values, and clinicopathologic parameters

All patients were categorized into high LY group (≥1.45 × 10^9^/L), low LY group (<1.45 × 10^9^/L), high NLR group (≥3.029), and low NLR group (<3.029) before radiotherapy, and high LY group (≥0.35 × 10^9^/L), low LY group (<0.35 × 10^9^/L), high NLR group (≥5.071), and low NLR group (<5.155) during treatment based on threshold value obtained through ROC analysis (<5.071). As shown in Tables 2 and 3, we compared the pathological and clinical characteristics between patients categorized by LY and NLR, respectively. We found no correlation between age and LY or NLR. However, the values of LY and NLR before and after radiotherapy were significantly correlated with FIGO staging (*p* = 0.036, 0.046, 0.013, and 0.042, respectively). Pathological type was correlated with the value of LY before radiotherapy (*p* = 0.026), and lymph node status was correlated with the values of LY and NLR before radiotherapy (*p* = 0.021 and 0.025, respectively) and the value of LY during radiotherapy (*p* = 0.041).

**TABLE 2:** The relationship of values before radiotherapy of LY and NLR with clinical and pathological parameter in CC patients.

Variables	No. patients (*n* = 202)	LY (×10^9^)		χ^2^	*p*	NLR		χ^2^	*p*
		<1.45	≥1.45			<3.029	≥3.029		
Age									
<50	101	37	64	0.189	0.772	64	37	1.440	0.294
≥50	101	40	61			72	29		
FIGO stage									
Stage I	127	41	86	4.938	0.036	94	33	6.957	0.013
Stage II	75	36	39			42	33		
Pathology									
SqCC	177	62	115	5.791	0.026	121	56	0.696	0.495
Other	25	15	10			15	10		
Lymph node									
N(−)	150	50	100	5.657	0.021	108	42	5.785	0.025
N(+)	52	27	25			28	24		

**TABLE 3:** The relationship of values during radiotherapy of LY and NLR with clinical and pathological parameter in CC patients.

Variables	No. patients (*n* = 202)	LY (×10^9^)		χ^2^	*p*	NLR		χ^2^	*p*
		<0.35	≥0.35			<5.071	≥5.071		
Age									
<50	101	31	70	3.174	0.105	51	50	0.079	0.888
≥50	101	20	81			49	52		
FIGO stage									
Stage I	127	26	101	4.132	0.046	70	57	4.311	0.042
Stage II	75	25	50			30	45		
Pathology									
SqCC	177	46	131	0.416	0.628	91	86	2.082	0.200
Other	25	5	20			9	16		
Lymph node									
N(−)	150	32	118	4.730	0.041	27	25	0.164	0.748
N(+)	52	19	33			73	77		

### 3.3 Prognostic value analysis of LY and NLR

**FIGURE 2:**
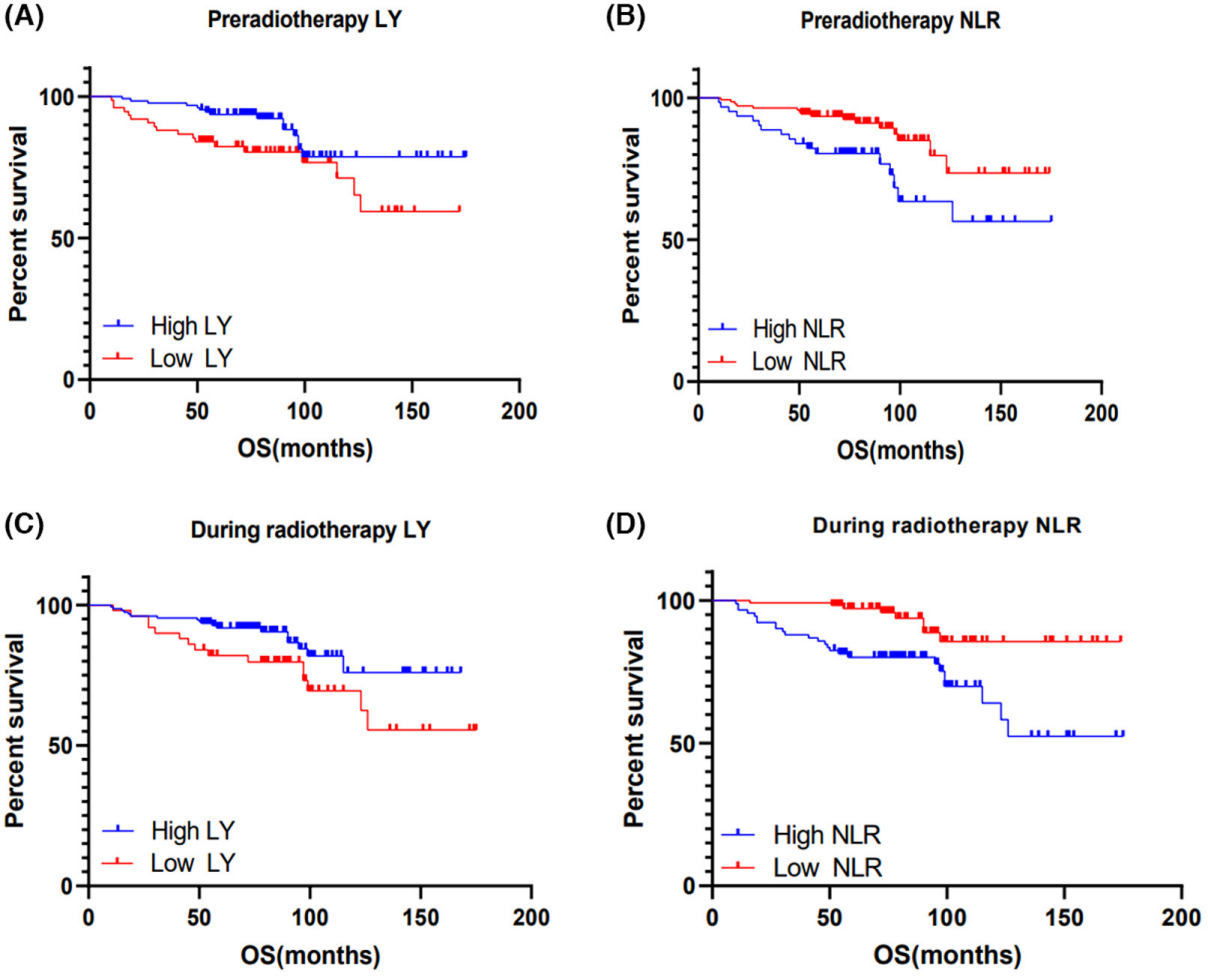
Kaplan–Meier survival curves for overall survival (OS) of cervical cancer patients grouped by LY and NLR cut‐off points. OS based on (A) pre radiotherapy LY, (B) pre radiotherapy NLR, (C) during radiotherapy LY, and (D) during radiotherapy NLR.

After grouping all 202 patients based on the ROC curve's cutoff value, a survival analysis was carried out to assess the predictive significance of LY and NLR. The cutoff points of OS predicted by pre radiotherapy values of LY and NLR were 1.45 × 10^9^/L and 3.029, respectively. As illustrated in Figure 2A,B patients with higher LY group (>1.45 × 10^9^/L) and lower NLR levels (≤3.029) had significantly better OS in CC (*p* = 0.042 and 0.008, respectively). Similarly, during radiotherapy, the cutoff points of OS predicted by LY and NLR values were 0.35 × 10^9^/L and 5.071, respectively. The OS of CC patients was also significantly improved in the higher LY (>0.35 × 10^9^/L) and lower NLR (≤5.071) groups (*p* = 0.042 and 0.001, respectively), as shown in Figure 2C,D. These results suggest that both pre radiotherapy and during radiotherapy values of LY and NLR are valuable prognostic factors for CC patients.

### 3.4 Analysis of relationship between LY, NLR values, and relapse patterns

Out of the 202 patients included in this study, a total of 62 patients experienced local or distant relapse. For patients with multiple relapses, the relapse pattern after the initial radiation therapy was selected to be included to analyzed. As shown in Table 4, we further analyzed the correlation between the LY or NLR values before or during radiotherapy and different relapse patterns. Our analysis revealed that a lower LY value before radiotherapy and higher NLR value during radiotherapy were significantly associated with a poorer relapse pattern (*p* = 0.041 and 0.035, respectively). However, no relationship was found between the NLR value before radiotherapy or LY value during radiotherapy and recurrence pattern (*p* = 0.071 and 0.303, respectively).

**TABLE 4:** The relationship between LY/NLR values before or during radiotherapy and relapse patterns in CC patients.

Variables	No. patients (*n* = 62)	Relapse		χ^2^	*p*
		Local	Distant		
Preradiotherapy					
Lower LY	33	15	18	4.607	0.041
Higher LY	29	21	8		
Lower NLR	31	14	17	4.239	0.071
Higher NLR	31	22	9		
During radiotherapy					
Lower LY	32	21	11	1.552	0.303
Higher LY	30	15	15		
Lower NLR	25	19	6	5.534	0.035
Higher NLR	37	17	20		

### 3.5 Independent prognostic indicators for PFS and OS

As the Kaplan–Meier survival analysis indicated the significant impact of LY and NLR on the survival of CC patients, COX regression analysis was carried out to assess their prognostic value in conjunction with other factors. The findings of univariate and multivariate analyses examining the potential predictors related to PFS are exhibited in Tables 5 and 6. The univariate analysis suggested that lymph node status, FIGO stage, pre and during radiotherapy values of LY and NLR were linked to PFS and OS, while the histological type and treatment strategy were connected to PFS but not OS. Multivariate analysis further demonstrated that FIGO stage I (*p* = 0.000), pathological type of SqCC (*p* = 0.006), absence of lymph node metastasis (*p* = 0.002), concurrent chemoradiotherapy (*p* = 0.003), higher lymphocyte values during radiotherapy (*p* = 0.008), and lower NLR values pre radiotherapy (*p* = 0.000) were independent predictors for improving PFS. In addition, FIGO stage I (*p* = 0.044), absence of lymph node metastasis (*p* = 0.011) and lower NLR values before (*p* = 0.042) and during radiotherapy (*p* = 0.019) were independent predictors for improving OS.

**TABLE 5:** Analysis of factors associated with progression‐free survival using univariate and multivariate methods.

	Univariate		Multivariate	
	HR (95% CI)	*p*‐value	HR (95% CI)	*p*‐value
Age (years)	1.416 (0.856–2.343)	0.176	–	–
<50				
≥50				
FIGO stage	2.971 (1.792–4.924)	0.000	2.805 (1.650–4.766)	0.000
Stage I				
Stage II				
Pathology	2.272 (1.206–4.283)	0.011	2.785 (1.335–5.813)	0.006
SqCC				
Other				
Lymph node metastasis	2.629 (1.604–4.310)	0.000	2.427 (1.392–4.232)	0.002
No				
Yes				
Treatment strategy	1.727 (1.045–2.853)	0.033	2.362 (1.349–4.136	0.003
CCRT				
EBRT				
During treatment LY	0.006 (0.001–0.046)	0.000	0.036 (0.003–0.420)	0.008
During treatment NLR	1.106 (1.067–1.146)	0.000	1.030 (0.983–1.080)	0.216
Pretreatment LY	0.233 (0.123–0.444)	0.000	0.824 (0.406–1.675)	0.593
Pretreatment NLR	1.168 (1.117–1.220)	0.000	1.146 (1.070–1.226)	0.000

**TABLE 6:** Analysis of factors associated with overall survival using univariate and multivariate methods.

	Univariate		Multivariate	
	HR (95% CI)	*p*‐value	HR (95% CI)	*p*‐value
Age(years)	1.342 (0.675–2.669)	0.402	–	–
<50				
≥50				
FIGO stage	2.391 (1.197–4.776)	0.014	2.074 (1.021–4.215)	0.044
Stage I				
Stage II				
Pathology	2.067 (0.896–4.766)	0.089	–	–
SqCC				
Other				
Lymph node metastasis	2.781 (1.370–5.244)	0.004	2.558 (1.239–5.281)	0.011
No				
Yes	1.101(0.541–2.240)	0.791	–	–
Treatment strategy				
CCRT				
EBRT				
During treatment LY	0.066 (0.005–0.840)	0.036	0.939 (0.053–16.741)	0.966
During treatment NLR	1.090 (1.037–1.145)	0.001	1.066 (1.002–1.134)	0.042
Pretreatment LY	0.382 (0.168–0.869)	0.022	0.968 (0.394–2.376)	0.943
Pretreatment NLR	1.131 (1.067–1.199)	0.000	1.101 (1.016–1.193)	0.019

## 4 DISCUSSION

Prior research has proposed that the prognosis of CC is highly linked with factors such as tumor stage, pathological type, degree of differentiation, and the presence of lymph node metastasis.^16^ However, obtaining these results often requires multiple imaging and biopsies, emphasizing the need to identify clinically accessible prognostic indicators of CC. In this retrospective study, we examined the predictive value of the minimum LY value and corresponding NLR value during radiotherapy on the prognosis of CC patients. We also included LY and NLR values pre radiotherapy for comparative analysis. Our results indicated that both LY and NLR values during and before radiotherapy were linked with the prognosis of CC patients. Patients with lower LY and higher NLR values during or before radiotherapy had a poorer prognosis. Additionally, lower LY and higher NLR values during radiotherapy were both independently linked with a poorer OS and PFS, respectively. While previous researches have revealed the relevance of LY and NLR to the prognosis of CC patients, most have focused on pre and posttreatment values, with little attention given to the prognostic value of various parameters during treatment. Our study highlights the importance of considering LY and NLR values during radiotherapy in predicting the prognosis of CC patients.

The materiality of immunotherapy in the treatment of tumors has been gaining more attention due to the close association between the human immune system and tumor progression. Recently, scholars have shown interest in combining immunotherapy with traditional chemoradiotherapy to treat CC. For instance, a recent study demonstrated that using ipilimumab and chemoradiotherapy together improved PFS and OS in CC patients with lymph node metastases.^17^ Lymphocytes play a significant role in the immune system's recognition and elimination of new tumor cells, especially as the introduction of the concept of immune surveillance.^18^ Therefore, the correlation of LY and its related indicators to clinicopathologic parameters and prognosis of tumors has attracted more and more attention. Previous studies have demonstrated an association between pretreatment LY values and FIGO stage, as well as lymph node metastasis, in CC patients.^19^, ^20^ In this study, we found that both LY or NLR levels before and during radiotherapy were associated with various clinicopathologic features, including FIGO stage, pathological type, and lymph node status. Higher LY counts have been linked to a better prognosis for various malignant cancers, including breast cancer^21^ and non‐small‐cell lung cancer.^22^ Likewise, higher LY counts are strongly associated with better outcomes for CC patients.^19^ Previous researches have shown that lower LY values before treatment have been correlated with worse OS and PFS in CC patients, and persistent lymphopenia after radiotherapy is a risk factor for poorer prognosis in CC patients.^23^ Patients with increased LY after surgery for CC have significantly longer PFS than patients with decreased LY.^24^ Our study suggests that higher LY counts before and during radiotherapy are linked with better PFS and OS in CC patients. Furthermore, multivariate analysis indicated that higher LY values during radiotherapy were an independent predictor of better PFS. Few studies have examined the effect of LY counts during radiotherapy on CC patients' prognosis until now. The aforementioned studies have suggested that the lower value of LY was linked with a higher incidence of recurrence, metastasis, and mortality in patients with CC posttreatment. However, few studies have specifically analyzed the relationship with relapse patterns. Fattorossi et al.'s research have demonstrated that the proportion of lymphocyte subsets was significantly correlated with the distance to lymph node metastasis of CC.^25^ In this study, we found that a lower LY value before radiotherapy was associated with a higher incidence of distant metastases, while a higher LY value was linked to a greater frequency of local metastases.

In recent years, a growing body of literature has shown that an elevated NLR value is linked with the progression and metastasis of various tumors.^26^, ^27^, ^28^ NLR is a measure utilized to evaluate the equilibrium between the pro‐tumor inflammatory response and the anti‐tumor immune response, by calculating the proportion of neutrophil to lymphocyte ratio. An increase in NLR may indicate an increase in pro‐tumor inflammatory response and a decrease in anti‐tumor immune response.^29^

Zhu et al.'s study demonstrated a correlation between pretreatment NLR values and FIGO stage and parametrial involvement in CC, although no association with lymph node metastasis was observed.^30^ Similarly, Zhang et al.'s study suggested that the NLR values could effectively predict lymph node metastasis in patients with gastric cancer.^31^ A meta‐analysis has reported that higher values of NLR were significantly associated with lymph node metastasis in CC patients.^32^ In this study, we found that pre radiotherapy NLR values were significantly linked to both FIGO stage and lymph node metastasis in CC patients. Previous research has reported that a higher NLR value is linked with poorer survival outcomes in CC patients. Lee et al.^33^ investigated the predictive effect of changes in NLR before and after concurrent chemoradiotherapy on the prognosis of patients with advanced CC and found that changes in NLR before and after treatment, especially increases in NLR, were prognostic factors that could influence the risk level for patients with CC independently. Despite the predictive significance of changes in NLR value before and after treatment for CC patient prognosis, few studies have concentrated on the predictive significance of NLR value during radiotherapy. Our study confirmed that a higher NLR value during radiotherapy might be an important adverse prognostic factor for CC. This finding could be clarified by the mechanism of NLR's involvement in the inflammatory response of tumor patients, as described above. Meanwhile, we conducted further analysis and discovered a correlation between the NLR value during treatment and the recurrence pattern of CC patients. It was observed that higher NLR was more likely to be associated with distant recurrence than lower NLR. However, few studies have focused on the relationship between NLR values and relapse patterns.

Despite the significant predictive value of changes in LY and NLR values before and after CC treatment, there have been few studies focusing on the predictive significance of LY and NLR values during radiotherapy. This study aimed to address this gap by analyzing the correlation between LY and NLR values before and during radiotherapy, clinicopathology factors, and prognostic value in a cohort of 202 CC patients who underwent surgery. Although previous studies and this study have demonstrated the correlation between LY and NLR values and tumor clinicopathological factors and prognosis, the underlying mechanisms have not been fully elucidated. Current research suggests that it could be primarily explained by the function of LY subpopulations. CD8^+^ T cells may inhibit tumor growth and induce tumor cell apoptosis through cytotoxic effects, while CD4^+^ T cells can secrete cytokines necessary for the growth and proliferation of CD8^+^ T cells.^34^, ^35^ The synergistic action of the two subpopulations can produce a stronger antitumor effect than either subpopulation alone. A recent study has discovered that CD4^+^ T cells could control tumor growth and progression by secreting th1 cytokines to reactivate tumor cell senescence.^36^ Tumor‐associated neutrophils have been identified as key mediators in angiogenesis, tumor progression, evil transformation, and the regulation of antitumor immunity.^37^ Study have shown that neutrophils can inhibit the activity of LY and T cell responses, suppressing the immune system.^38^ Neutrophils may also secrete cytokines, including growth factors and chemokines, which contribute to an inflammatory microenvironment that promotes angiogenesis, tumor growth and tumor cell migration.^39^ It has been observed that neutrophils have the ability to release vascular endothelial growth factor (VEGF), which facilitates tumor vascularization, promoting tumor growth and progression.^40^ Studies have also revealed that elevated NLR is linked with a decrease in important anti‐tumor immune cells in the tumor microenvironment.^41^ Tumor‐associated neutrophils have been shown to promote the dislocation of tumor cells and endothelial cells, as well as enhance angiogenesis through enzymatic action. These effects eventually promote tumor growth and metastasis.^42^ A study by Han et al. revealed that in the presence of a lower NLR, there was a higher infiltration of CD8^+^ T cells and other immune cells into the tumor microenvironment, leading to improved therapeutic outcomes.^43^ Previous research has shown that patients with higher NLR exhibit a lower response to local radiotherapy.^44^ In general, a decrease in LY or an increase in NLR indicates a decrease in antitumor effect or an increase in pro‐tumor effect. The complex interaction between the immune system and inflammatory response ultimately leads to the deterioration of tumor‐related clinicopathologic factors and prognosis.

While our study provides important insights into the prognostic significance of lymphocyte count and NLR in CC patients undergoing radiotherapy, there are several limitations of the research that must be acknowledged. First, the number of participants in this study was limited, and all of them were enrolled from a single hospital, which could constrain the overall applicability of our results. Therefore, larger multicenter, future researches are needed to validate our results. Second, our study was retrospective, which may introduce selection bias. Future studies should consider using a prospective study design to minimize such bias. Third, the hematological results were derived from the detection of peripheral blood samples, which may not completely represent the immune status of the tumor microenvironment. Further studies are required to explore the relationship between the peripheral blood and tumor microenvironment and the predictive value and mechanism of changes in lymphocyte count and NLR to PFS and OS in CC patients.

## 5 CONCLUSION

In summary, our study revealed that the minimum LY level and corresponding NLR values during radiotherapy hold significant predictive value for the prognosis of CC patients. Additionally, a higher LY value during radiotherapy was discovered to be linked independently with poorer PFS, while a higher NLR value during radiotherapy was linked to poorer OS. As both LY and NLR are routine detection indexes in blood examinations, their values can be dynamically monitored to predict patient prognosis. Furthermore, the LY and NLR values during radiotherapy can serve as simple, convenient, and repeatable indicators for identifying potentially high‐risk patients. However, before this approach can be routinely applied in clinical practice, more large‐scale, multicenter prospective studies are needed to confirm its efficacy.

## AUTHOR CONTRIBUTIONS

**Mengli Zhao:** Conceptualization (equal); writing – original draft (lead). **Zhongrong Gao:** Visualization (equal). **Xiaowei Gu:** Data curation (equal); visualization (equal). **Xiaojing Yang:** Data curation (equal). **Shanshan Wang:** Data curation (supporting). **Jie Fu:** Project administration (equal); writing – review and editing (lead).

## FUNDING INFORMATION

This work was supported by the Science and Technology Project of Shanghai Municipal Science and Technology Commission (No. 22Y31900500).

## CONFLICT OF INTEREST STATEMENT

The authors declare that the research was conducted without any conflict of interest.

## ETHICS STATEMENT

The study was conducted in accordance with the Declaration of Helsinki and was approved by the Ethics Committee of Shanghai Sixth People's Hospital (protocol code 2022‐KY‐149(K) and 2022‐10‐20 of approval).

## CONSENT

Not applicable.

## Data Availability

Data are available upon reasonable request.
